# Use of staging for sex cord stromal tumours

**DOI:** 10.1097/CCO.0000000000000860

**Published:** 2022-07-16

**Authors:** Serena Negri, Tommaso Grassi, Robert Fruscio

**Affiliations:** a Clinic of Obstetrics and Gynecology, Department of Medicine and Surgery, University of Milan-Bicocca, Milan; b Unit of Gynecological Surgery, San Gerardo Hospital, ASST Monza, Italy

**Keywords:** granulosa cell tumour, Sertoli-Leydig cell tumour, sex cord stromal tumours, surgical staging

## Abstract

Sex cord-stromal tumours (SCSTs) are rare ovarian cancers. As in the literature, only small case series or case reports are published, gathering solid evidence about their management is challenging. Surgery plays a pivotal role, and accurate staging is one of the most important prognostic factors. This review focuses on the current evidence for surgical staging in the management of SCSTs.

**Recent findings:**

Staging procedures have been inferred by epithelial ovarian cancers; however, they are often only partially performed, and most SCSTs therefore end up incompletely staged, raising the issue of the need for restaging or further treatments. In addition, some parts of the staging procedure have been questioned over the years, and lymphadenectomy is now considered unnecessary for SCSTs.

The generally favourable prognosis of SCSTs, the introduction of minimally invasive surgery and fertility-sparing approaches is empowering the question of which staging procedures are beneficial for these patients. We reviewed the role of each staging procedure proposed by the guidelines in light of new scientific updates.

**Summary:**

Surgical staging should always be performed. It includes peritoneal samplings (peritoneal washing, multiple peritoneal biopsies, omental biopsy and biopsy of any suspicious area), whereas lymphadenectomy could be omitted. Laparoscopy may be considered a feasible approach.

## INTRODUCTION

Sex cord-stromal tumours (SCSTs) are nonepithelial ovarian neoplasms containing a pure or mixed combination of sex cord (granulosa and Sertoli cells) and stromal cells (fibroblasts, theca and Leydig cells). They represent 3–7% of all ovarian tumours, with an estimated incidence of 2.1 per 1 million women in Europe [1–3] and include a complex variety of different histotypes, both benign and malignant, recently categorized by the WHO classification into three major groups: pure stromal, pure sex cord and mixed sex cord-stromal tumours [4]. This review will mainly focus on the most frequent malignant SCSTs: granulosa cell tumours (GCTs) and Sertoli-Leydig cell tumours (SLCTs).

GCTs are the most frequent form of malignant SCSTs. They are classified as pure sex cord tumours and further divided into an adult and a juvenile form. Adult granulosa cell tumours (AGCTs) are considered low-grade malignant tumours, and they account for up to 95% of GCTs. Their mean age of onset is around 50–55 years, involving typically perimenopausal and postmenopausal women. Juvenile GCTs (JGCTs) are rare ovarian neoplasms, but they represent the most common type of SCSTs in children and adolescents, with a mean age of onset of 13 years [5]. The SLCTs are mixed SCSTs, whose clinical behaviour depends on the degree of differentiation and stage [6]. They are very uncommon and in 75% of cases arise in women under 40 years of age [3].

The majority of malignant SCSTs generally have an indolent course and a favourable prognosis mainly because they are diagnosed at an early clinical stage [3,7,8]. Stage is one of the main prognostic factors in SCSTs, and it is essential to determine the need for adjuvant treatment. In AGCTs, the 5-year overall survival (OS) rate was 98.7% in stages I-II and 75% in stages III-IV [9], while in SLCTs was estimated to be up to 60–84.8% for stages I-II and 33–35% for stages III-IV [10^▪▪^].

The advances in imaging techniques have greatly improved the clinical staging of disease and the monitoring of recurrence [1,11–14,15^▪^]. However, surgery is still the mainstay of staging and treatment for malignant SCSTs. It is important to remember that a fertility-sparing approach may be considered in young women with malignant SCSTs confined to the ovary [1,3,16–23].

This review will discuss each staging procedure proposed in the management of SCSTs of the ovary, focusing on the new scientific updates. 

**Box 1:**
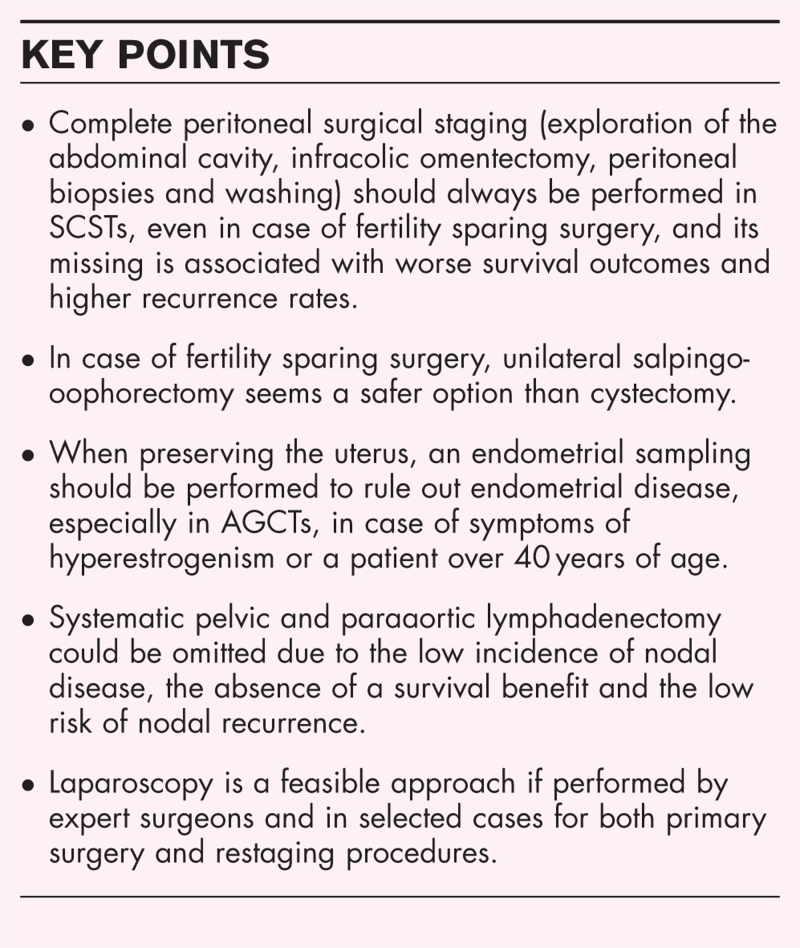
no caption available

## CLINICAL STAGING

When approaching a newly diagnosed ovarian mass, imaging procedures are fundamental.

GCTs usually present as unilateral large masses, with a multicystic with solid components or solid heterogeneous appearance on ultrasound and CT, mirrored by a ‘sponge-like’ appearance in MRI [11–12]. They are rarely associated with peritoneal carcinomatosis [11,14]. Uterine changes and endometrial thickening are better detected on ultrasound or MRI than CT [14]. As most GCTs have low to moderate FDG-avidity, PET is not always reliable in evaluating the extension of disease or monitoring for recurrence [14,15^▪^].

SLCTs are frequently unilateral solid masses, but they lack a characteristic appearance on imaging [11].

## SURGICAL STAGING

Surgery is the mainstay of staging and treatment for malignant SCSTs. International guidelines suggest peritoneal staging (exploration of the abdominal cavity, infracolic omentectomy, peritoneal biopsies and washing); endometrial biopsy or hysterectomy; and no retroperitoneal staging required.

### Peritoneal staging

Staging procedures in apparent stage I disease include peritoneal fluid examination or washing, infracolic omentectomy and random peritoneal biopsies (diaphragmatic peritoneum, paracolic gutters, pelvic peritoneum) [1,24^▪▪^]. The peritoneal spread of the disease is the most frequent in SCSTs, as the peritoneum is the main localization of disease in advanced stage or relapsed cases.

In a retrospective series of patients with AGCTs [19], 12% (13/106) had an advanced stage at the time of the diagnosis. They all had a peritoneal disease diffusion, while no lymph nodal disease was found. Interestingly, two of these 13 patients had a clinical early-stage disease, but they were upstaged thanks to the complete peritoneal surgical staging. In another retrospective multicentre study, Thrall *et al.*[25] evaluated the pattern of spread of 87 SCSTs (71 AGCTs, 11 SLCTs, one JGCT and four mixed or unclassified SCSTs). FIGO stage was more than stage I in 10 cases (six stage II, three stage III and one stage IV). In two patients with stage II disease and one with stage III, macroscopic pelvic or abdominal disease was found at the time of surgery. However, in the other four patients with stage II and two with stage III, the disease was microscopically found by peritoneal biopsies in the cul de sac, pelvic sidewall, uterosacral ligament, diaphragmatic peritoneum or omentum. No lymphatic dissemination of disease was found at the time of diagnosis. As reported, both GCTs and SLCTs were included in the study, but the histology of patients with advanced-stage disease was not specified.

Therefore, performing peritoneal sampling is crucial for the correct staging of SCSTs, especially considering the poor prognosis related to advanced-stage disease [9,10^▪▪^]. Moreover, the persistence of residual disease after surgery, which can be missed if staging procedures are not performed, seems to be a negative prognostic factor affecting the recurrence rate [26].

Another reason to perform peritoneal staging is that recurrence is mainly located in the peritoneal cavity, suggesting this is the favourite route of spread and can be missed during the primary surgery if staging is not performed [26–29]. In a series of 35 recurrent GCTs [26], the site of the first recurrence was pelvic and/or abdominal in 26 cases (74%), pelvic/abdominal and lymph-nodal in seven (20%) and lymph-nodal only in two cases (6%). Recently, Nef *et al.*[28] published a series of 85 relapsed SLCTs, and for 75 of them, the location of the relapse was reported. In 62 patients (83%), the relapse was pelvic and/or abdominal. Only five women (7%) had a relapse involving lymph nodes, and it was always associated with a pelvic/abdominal recurrence. Eight cases (10%) had relapses involving a distant organ. As one of the most common recurrent disease sites is the peritoneal cavity for both GCTs and SLCTs, often in a multifocal pattern [28,30], some authors suggest that it could be a missed localization at the first surgery [19,31].

### Hysterectomy and endometrial sampling

SCSTs can frequently present with signs of abnormal hormonal production, such as menstrual irregularities, abnormal bleeding or, less frequently, virilization.

ACGTs are typically oestrogen-secreting ovarian cancers (70% of cases [32,33]). The resulting hyperestrogenism, especially if prolonged and unopposed, can lead to endometrial hyperplasia and endometrial cancer in 20–60 and 1–20% of cases, respectively [7,17,34–38] (Table 1). The wide range of incidence may derive from different histological criteria used to define hyperplasia and endometrial cancer. Some authors suggest that endometrial cancer incidence is less than 5% if strict criteria are applied [3,7,39]. JGCTs can manifest with hyperestrogenism and pseudoprecocity, but no studies suggest an association with endometrial pathologies. An increased incidence of endometrial carcinoma (1.1–19%) and endometrial hyperplasia (3–50%) was reported in thecomas, especially in postmenopausal women [34,35]. GCTs are rarely associated with androgen production, whereas hyperandrogenism is the most common presentation in SLCTs. However, rare cases of endometrial hyperplasia concomitant to SLCTs have been reported in the literature [40,41], and one case of endometrial cancer has been described [42].

**Table 1:** Prevalence of endometrial disease in granulosa cell tumors

	Endometrial histology		
	Hyperplasia		Cancer
	Typical	Atypical	
Gusberg and Kardon [34] *n* = 69 GCTs	13%	42%	22% (+ 5% in situ)
Stenwig *et al*. [7] *n* = 64 GCTs	64%	1.6%	3.1%
Evans *et al*. [35] *n* = 76 GCTs	55%		13%
Ayhan *et al*. [17] *n* = 80 Adult GCTs	60%		1.2%
Lee *et al*. [18] *n* = 68 Adult GCTs	17.6%	5.9%	2.9%
Thrall *et al*. [25] *n* = 71 Adult GCTs	0%	0%	6%
Park *et al*. [19] *n* = 106 Adult GCTs	15.1%		0.9%
Van Meurs *et al*. [36] *n* = 1031 GCTs	16.5%	9%	5.9%
Ottolina *et al*. [37] *n* = 140 Adult GCTs	22%		5.7%
Bergamini *et al*. [38] *n* = 223 Stage I adult GCTs	NA	6.7%	4.5%

GCTs, granulosa cell tumours.

The standard surgical approach for epithelial ovarian cancer includes hysterectomy as a part of the staging. Anyway, as most SCSTs occur in young patients, fertility-sparing surgery (FSS, unilateral salpingo-oophorectomy) and complete intra-abdominal/peritoneal staging can be considered if the disease is confined to the ovary [1,3,22].

When preserving the uterus in adult patients with GCTs, endometrial sampling at the time of diagnosis is recommended to exclude endometrial hyperplasia and/or endometrial cancer [1,3,24^▪▪^].

If no abnormalities are found, the lifetime risk of developing one is similar to the general population and is often associated with recurrence of GCT [36].

As the incidence of endometrial abnormalities has been shown significantly associated with the presence of symptoms (35 vs. 19.2%) and age more than 40 years (25.9 vs. 3.3%), some authors suggest performing endometrial sampling only in these cases, whereas an ultrasound evaluation of the endometrium thickness before surgery is recommended in asymptomatic young women [37].

### Salpingo-oophorectomy or cystectomy

Bilateral salpingo-oophorectomy is the indicated approach in SCSTs when there is no need or wish to preserve fertility and in advanced-stage disease.

In stage I disease, conservative surgery could be considered [1,3,13,16–23], as SCSTs are mostly unilateral, with an estimated bilateral involvement in only 2–8% of GCTs [20] and 1.5–2% of SLCTs [8].

When choosing a fertility-sparing approach in stage I AGCTs, unilateral salpingo-oophorectomy (USO) has proven to be a better option than cystectomy [20,31]. USO showed no significant difference in OS and disease-free survival (DFS) compared with radical surgery (bilateral salpingo-oophorectomy with or without hysterectomy) [20,31]. On the contrary, patients undergoing cystectomy showed a worse DFS compared with USO and radical surgery, with relapses in 76.9–85.7% of cases [20,31]. The most frequent sites of relapse following cystectomy were the same ovary (30–100%) and the contralateral ovary (40%). If cystectomy is performed, reoperation to remove the remaining adnexa is recommended, as it has shown to improve prognosis [20,31]. In addition, incomplete peritoneal staging was more frequent in the FSS group in both the aforementioned retrospective studies, and it has been associated with significantly worse DFS [31] and general worse prognosis [20].

Cystectomy might be well tolerated in JGCTs [13,43]; however, a recent review described a recurrence rate of 25%, with one of four patients relapsing after 3 months in the previously affected ovary [44^▪▪^].

USO is recommended for young patients with stage IA SLCTs [1,45], and might be considered an option even in selected cases of advanced stage or recurrence [1,46].

In case of conservative surgery for a SCST, a meticulous macroscopic evaluation of the contralateral adnexa is recommended, and biopsies of suspicious areas should be performed, while random ovarian biopsies are not necessary [13,43,47].

The use of completion of surgery at the conclusion of childbearing or after 40 years old seems reasonable but is still controversial [3,47,48]. As the recurrence rate is around 20–30% and salvage therapies have shown to be effective, some authors suggest delaying radical surgery until the time of recurrence [31].

### Retroperitoneal staging: Lymphadenectomy

Systematic pelvic and paraaortic lymphadenectomy is part of the recommended staging procedure for early-stage epithelial ovarian cancers and it was initially proposed in the management of SCSTs as well.

However, in a recent study, the incidence of lymph nodes metastases was 3.3 and 4.1% in patients with stage I-IV GCTs and SLCTs, respectively [10^▪▪^]. When stratified by stage, the incidence of lymph nodes metastasis is around 0–4.5% in early-stage SCSTs [17,19,30,42,49–55] (Table 2), much lower than in apparent stage I-II epithelial ovarian cancers (14.2%) [56]. In advanced-stage SCSTs, fewer data are available. According to two recent studies, the incidence of positive lymph nodes increases with the stage of disease in GCTs: 13.3% in stage II, 23.3–26.7% in stage III and 26.9% in stage IV [55,57]. Therefore, more data are needed for the possible role of a lymph node dissection in advanced-stage GCTs. To the best of our knowledge, no data are available for advanced-stage SLCTs.

**Table 2:** Incidence of positive lymph nodes in Sex cord-stromal tumors

	Histological type		Stage I-II		Stage III-IV	
			Number of patients with positive LN on number of patients who performed LND		Number of patients with positive LN on number of patients who performed LND	
	Granulosa	Nongranulosa	Granulosa	Non-Granulosa	Granulosa	Non-Granulosa
Abu-Rustum *et al*. [50] (2006)	68 (100%) –64 AGCTs –4 JGCTs	–	0/13			
Ayhan *et al*. [17]	80 (100%)	–	7/80 (8.8%)			
Brown *et al*. [30]	205 (79.7%) –178 AGCTs –27 JGCTs	52 (20.3%) –31 SLCTs	0/37 (30 AGCTs, 7 JGCTs)	0/12 (5 SLCTs)	0/8 (6 AGCTs, 2 JGCTs)	0/1 (0 SLCTs)
Park *et al*. [19]	106 (100%) AGCTs	–	0/25	–	0/2	–
Karalök *et al*. [51]	10 (100%) JGCTs	–	0/5	–	–	–
Nasioudis *et al*. [52]	954 (82.5%) –945 AGCT –9 JGCT	202 (17.5%)	15/473 (3.2%)	4/99 (4%)	–	–
Kuru *et al*. [53]	151 (100%) AGCTs	–	6/134 (4.5%)			
Cheng *et al*. [54]	50 (69.4%) –39 AGCT –11 JGCT	22 (30.6%) –18 SLCT	0/28		0/6	
Ebina *et al*. [55]	1426 (100%)	–	6/207 (0.3%)	–	4/15 (26.7%)	–
Wang *et al*. [44^▪▪^]	35 (100%) JGCTs	–	0/14	–	–	–

AGCT, adult granulosa cell tumour; JGCT, juvenile granulosa cell tumour; SCSTs, sex-cord stromal tumours; SLCT, Sertoli-Leydig cell tumour.

A few retrospective series investigated the possible role of lymphadenectomy in SCSTs [52,54,58]. Lymphadenectomy did not improve DFS [44^▪▪^,^58^] nor OS [44^▪▪^,^52^,^54^,^58^]. In contrast, it was significantly associated with increased postoperative morbidities, such as longer hospital stay, increased wound infection rate and decreased serum haemoglobin [58]. However, the vast majority of patients in these studies had an early-stage disease.

At the time of the recurrence, the disease may involve lymph nodes. However, nodal recurrences are rare and seem unrelated to lymph nodes status at the time of diagnosis [30]. As they are more frequently associated with abdominopelvic sites of disease than isolated, they are thought to represent secondary spread of disease [30] rather than occult nodal metastasis as previously suggested [50].

In conclusion, due to the low incidence of nodal disease, the absence of a survival benefit and the low risk of nodal recurrence, the need for lymphadenectomy in SCSTs has been questioned in recent years and it is now not recommended by international guidelines [1,24^▪▪^,^45^,^59^]. Only suspicious nodes on imaging or during intraoperative staging procedures should be removed because the presence of nodal metastasis influences the need for postoperative chemotherapy. However, the role of adjuvant chemotherapy in SCSTs is currently debated, as some studies suggest limited survival benefits [60,61].

### Incomplete staging and restaging

Frequently, the diagnosis of SCST is made at the time of the pathology report when a conservative surgery without an adequate staging procedure has been performed. Incomplete staging can lead to underestimating the burden of the disease, a possible missed chance of treatment with second surgical procedure to achieve cytoreduction or with adjuvant chemotherapy, and, therefore, an increased risk of relapse [9,19,21,23,31].

Patients with early-stage AGCT and incomplete surgical staging have shown a worse DFS [9], whereas a low risk of recurrence is seen in patients with early-stage AGCT and adequate surgical staging [3,19]. In a recent review about early-stage JGCTs treated with USO or cystectomy, incomplete surgical staging is significantly associated with DFS and is the only risk factor for recurrence [44^▪▪^].

Secondary surgical staging can upstage a patient with a presumed early-stage SCST [25,31,44^▪▪^], and it should be performed to better assess the prognosis and management. The probability of upstaging may be influenced by the initial staging of the disease, as it seems more frequent in stage IC AGCTs than in stage IA (33 vs. 12%) [62^▪▪^].

Lymph node assessment could be omitted from the restaging procedure in the absence of clinical or radiological suspicion of nodal involvement [25,30,44^▪▪^].

As microscopic residual disease in the remaining ovary following a cystectomy has been reported [25,30], completion of surgery with salpingo-oophorectomy and adequate staging procedures should be always considered, even in young women willing to preserve fertility.

## THE SURGICAL APPROACH: LAPAROSCOPY VS. LAPAROTOMY

Minimally invasive surgery is associated with better outcomes, such as shorter hospital stay, faster recovery and fewer morbidities, and it has become increasingly more common in gynaecological surgeries. Its use in the treatment of early epithelial ovarian cancer has initially been discouraged by the fear of tumour rupture and incomplete staging [63–65]. However, some authors described successful laparoscopic approaches in SCSTs [63,66–68], suggesting that this surgical route could be a valid option if performed by expert surgeons in selected cases.

In a large retrospective study [38], laparoscopy has shown equivalent DFS and OS in stage I AGCTs. No difference was found in the rate of complete staging. Still, a higher incidence of tumour rupture was described, but it was not statistically significant, and not associated with worse survival rates or higher risk of port-site metastases.

Even though only small series and case reports are available, laparoscopy seems a well tolerated option also in JGCTs [43,44^▪▪^,^66^] and SLCTs [29,69].

As for restaging surgery, laparoscopy has shown to be a valid approach in AGCTs, with an upstaging rate of 19% compared with the 28% of laparotomy (not statistically different) [62^▪▪^].

Tertiary cytoreductive surgery by laparoscopy has been described [70] and the use of robot-assisted laparoscopy in recurrent AGCT has been recently reported [71], but the available evidence is still very limited.

## CONCLUSION

Staging procedures play a critical role in patients with SCSTs to determine the prognosis and the management. They should always include a complete peritoneal staging, whereas lymphadenectomy may be avoided due to the absence of any survival benefit and the low risk of nodal involvement. In case of FSS, USO is a safer option than cystectomy and an endometrial sampling is recommended especially in AGCTs. As incomplete staging is associated with worse survival outcomes, restaging is suggested. Laparoscopy is a feasible approach for both primary surgery and restaging procedures.

It is essential to note that as SCSTs are rare tumours, most evidence comes from retrospective studies and case reports, making it sometimes challenging to compare results and make definitive conclusions.

## Acknowledgements

*None.*

### Financial support and sponsorship

*None.*

### Conflicts of interest

*All the Authors disclose any possible conflict of interest. No financial support has been received for this work.*

## REFERENCES AND RECOMMENDED READING

Papers of particular interest, published within the annual period of review, have been highlighted as:

▪ of special interest
▪▪ of outstanding interest
